# Changes in Epithelial Ovarian Cancer Recurrence and Survival According to Treatment Paradigm Shifts

**DOI:** 10.1111/jog.70192

**Published:** 2026-02-12

**Authors:** Jeongyun Kim, Dong Hoon Suh, Kidong Kim, Jae Hong No, Yong Beom Kim

**Affiliations:** ^1^ Department of Obstetrics and Gynecology Seoul National University College of Medicine Seoul South Korea; ^2^ Department of Obstetrics and Gynecology Seoul National University Bundang Hospital Seongnam South Korea

**Keywords:** bevacizumab, cytoreductive surgery, ovarian cancer, poly(ADP‐ribose) polymerase inhibitors, treatment effectiveness

## Abstract

**Aim:**

To evaluate oncologic outcomes in patients with epithelial ovarian cancer (EOC) amid evolving surgical and systemic therapy paradigms.

**Methods:**

This retrospective cohort study included patients diagnosed with EOC from June 2003 to December 2020 at a single tertiary center, grouped by diagnosis period. Overall survival (OS) and progression‐free survival (PFS) were analyzed using the Kaplan–Meier and Cox regression analyses.

**Results:**

A total of 763 patients were classified as 2003–2008 (Group 1, *n* = 101), 2009–2013 (Group 2, *n* = 207), and 2014–2020 (Group 3, *n* = 455), reflecting changes in cytoreductive surgery and targeted therapies (bevacizumab and PARP inhibitors). Early‐stage diagnoses increased over time without statistical significance (Stage I–II: Group 1, 37.6% vs. Group 3, 46.6%; *p* = 0.200). Group 2 showed greater use of interval debulking surgery (IDS), higher complete cytoreduction rates, and more first‐line chemotherapy cycles (all *p* < 0.001). Group 3 represented the introduction of targeted therapies (*p* < 0.001 for both). IDS with residual (< 1 cm) was associated with poorer outcomes than complete/optimal primary debulking surgery (PDS) (hazard ratio 2.94, 95% confidence interval 1.5–5.8). Despite unchanged PFS, the 5‐year OS improved from 64.0% to 82.5% among patients with advanced‐stage disease (*p* = 0.024).

**Conclusions:**

Over two decades, with the advent of targeted therapies, complete cytoreduction (especially in PDS) has increased. Although the use of IDS also increased, residual disease (< 1 cm) after IDS was associated with poorer outcomes. While PFS remained unchanged, 5‐year OS significantly improved among patients with advanced‐stage disease diagnosed in the most recent period.

## 1 Introduction

Based on age‐standardized rates, ovarian cancer ranked eighth in incidence and fifth in mortality among women worldwide in 2022, while in South Korea in 2024, it ranked seventh and eighth, respectively [1, 2]. In South Korea, the age‐standardized incidence rate has increased from 5.1 to 7.8 per 100 000, reaching 3384 new cases in 2024, and the age‐standardized mortality rate has also increased from 2.3 to 2.5 per 100 000 compared with 2005 [3]. Despite being the leading cause of death from gynecologic malignancies in the country, the 5‐year relative survival rate improved from 60.4% in patients diagnosed in 1999–2005 to 64.1% in those diagnosed in 2013–2019 [4]. This improvement was observed across all stages and is likely attributable to earlier detection and advances in therapeutic strategies.

Over recent decades, continuous advances in ovarian cancer management have reshaped standard treatment, encompassing both surgical approaches and novel systemic therapies. The prognostic significance of residual disease volume after debulking surgery is well established, with aggressive cytoreduction advocated to achieve optimal debulking (residual tumor < 1 cm), particularly no gross residual disease [5, 6]. Regarding surgical timing, primary debulking surgery (PDS) is generally preferred; however, when PDS is deemed inappropriate, neoadjuvant chemotherapy followed by interval debulking surgery (IDS) is considered an alternative. Nevertheless, concerns about the oncologic outcomes of IDS remain, particularly with respect to chemoresistance. Similarly, the benefit of secondary cytoreductive surgery in recurrent platinum‐sensitive disease remains debated, with varying criteria for selecting candidates for complete cytoreduction [7, 8, 9].

Epithelial ovarian cancer (EOC), often diagnosed at an advanced stage with over 70% recurrence, is primarily managed with systemic therapy, typically carboplatin plus paclitaxel [10]. Although this regimen effectively controls primary and recurrent platinum‐sensitive disease, treatment failure or platinum resistance ultimately develops, leading to poor outcomes. To overcome the limitations of conventional cytotoxic agents, poly (ADP‐ribose) polymerase (PARP) inhibitors were developed and have demonstrated efficacy in improving progression‐free survival (PFS) and, in some cases, overall survival (OS) as maintenance therapies after platinum‐based chemotherapy—initially in recurrent settings in 2014 and, since 2018, in front‐line treatment [11, 12, 13, 14]. Additionally, bevacizumab, an anti‐VEGF monoclonal antibody, has shown efficacy in both primary and recurrent settings, particularly in platinum‐resistant disease [15].

In addition to these major developments, various efforts in both surgical and systemic approaches have aimed to improve outcomes in patients with ovarian cancer. In South Korea, such changes have been incorporated into the national health insurance system, facilitating their widespread adoption. Accordingly, in real‐world settings, we sought to examine changes in clinical practice while assessing their effectiveness in terms of survival and recurrence. Although these oncologic outcomes likely reflect the combined effects of multiple practices that were not individually addressed in this study, we focused on associations with recent findings most frequently reported in the current literature.

## 2 Methods

We retrospectively reviewed data from Seoul National University Bundang Hospital, South Korea, including patients with epithelial ovarian, fallopian tube, or primary peritoneal cancer who underwent primary treatment at this tertiary center between June 2003 and December 2020. The center opened in March 2003, with the first diagnosis included in this study occurring in June 2003. Patients were excluded if they had another malignancy requiring systemic therapy within 3 years of ovarian cancer treatment, were lost to follow‐up within 3 months of initial treatment, or were enrolled in any blinded clinical trial. Ethical approval was obtained from the Institutional Review Board (IRB No. B‐2404‐897‐106), which waived the requirement for informed consent due to the study's retrospective design and use of de‐identified data.

Data were extracted from the institutional electronic medical records, implemented in 2003, without using a separate research database. Variables included age at diagnosis, initial International Federation of Gynecology and Obstetrics (FIGO) stage, histologic type and grade, timing and extent of primary treatment, extent of secondary cytoreduction, number of first‐line chemotherapy cycles, use of targeted therapy, and dates of first recurrence, last follow‐up, and death. Histologic grade was evaluated using the surgical specimen or, if unavailable, biopsy data. Because the FIGO staging system has been revised over time, all patients were reclassified according to the 2014 version to align with current standards.

Patients were grouped by diagnosis year to reflect two major treatment trends: the proportion of complete or optimal debulking surgery (residual tumor < 1 cm) and the use of targeted therapy. Clinicopathologic characteristics, treatment approaches, and oncologic outcomes were compared across groups.

The primary outcome was OS to capture outcomes including recurrent treatments, and the secondary outcome was PFS before first recurrence. OS was defined as the time from treatment initiation to death or last follow‐up, and PFS as the time to first recurrence/progression or last follow‐up, whichever occurred first. Recurrence was determined from medical records by the treating physician at the time or, if unspecified, based on cross‐sectional imaging using the Response Evaluation Criteria in Solid Tumors (RECIST) and serum markers [16]. Associations between clinicopathologic factors and OS were evaluated using multivariable analysis. Also, temporal changes in oncologic outcomes were assessed separately by initial FIGO stage (I–II vs. III–IV).

Continuous variables were compared using the Kruskal–Wallis test after assessing normality, and categorical variables were analyzed with the chi‐square or Fisher's exact test, as appropriate. Post hoc pairwise comparisons were adjusted using Bonferroni correction. Clinicopathologic factors associated with OS were evaluated using a Cox proportional hazards model, reporting hazard ratios (HRs) and 95% confidence intervals (CIs). Variables with *p* < 0.1 in univariable analysis were included in the multivariable model. OS and PFS were analyzed using the Kaplan–Meier method and compared with the log‐rank test. All analyses were performed using SPSS version 29.0 (IBM Corp., Armonk, NY), except for stacked bar plots and survival curves, which were generated in R version 4.5.1 (R Foundation, Vienna, Austria) using the survival and survminer packages. Two‐sided *p* < 0.05 were considered statistically significant.

## 3 Results

Over 18 years, 836 patients received first‐line treatment at the center, of whom 763 (91.3%) were included in the study. Exclusions were due to another malignancy requiring systemic therapy within 3 years (*n* = 19), loss to follow‐up within 3 months of initial treatment (*n* = 20), and enrollment in a blinded trial (*n* = 34). Since 2009, more than 70% of patients (range, 74.4%–96.4%) consistently underwent optimal debulking surgery as first‐line treatment, compared with 40.0%–61.5% in earlier years (except 95.2% in 2007). Bevacizumab was consistently incorporated from 2014, and PARP inhibitors from 2018. Accordingly, patients were categorized into three groups by diagnosis year: 2003–2008 (Group 1, *n* = 101, 13.2%), 2009–2013 (Group 2, *n* = 207, 27.1%), and 2014–2020 (Group 3, *n* = 455, 59.6%).

Table 1 summarizes patient characteristics by diagnosis period. Age, initial FIGO stage, histologic type, and secondary cytoreduction showed no significant differences, while early‐stage diagnoses (I–II) increased from 37.6% in Group 1 to 46.6% in Group 3 (*p* = 0.200). Comparing Group 1 and Group 3, histologic grading (low and high: 9.9% and 30.7% in Group 1 vs. 24.0% and 71.2% in Group 3) and IDS (5.0% vs. 25.1%) became more frequent, with higher rates of complete cytoreduction among patients undergoing primary cytoreduction—either PDS or IDS (46.0% vs. 82.7%) (all *p* < 0.001). The proportion receiving more than six cycles of first‐line chemotherapy increased from 8.9% to 28.4% (*p* < 0.001). Use of targeted therapies also increased in Group 3, with bevacizumab rising from 1.0% to 27.9% and PARP inhibitor from 0% to 12.3% (both *p* < 0.001). Median follow‐up duration for Group 3 was 60.3 months, significantly shorter than Groups 1 and 2 (70.7 and 78.8 months, respectively; *p* < 0.001).

**TABLE 1:** Distributions of variables of interest by period of ovarian cancer diagnosis.

Variable	Total	Group 1 (2003–2008)	Group 2 (2009–2013) with optimal cytoreduction	Group 3 (2014–2020) with bevacizumab ± PARPi	*p*
	*N* = 763	*N* = 101 (13.2%)	*N* = 207 (27.1%)	*N* = 455 (59.6%)	
	No. of cases (%)	No. of cases (%)	No. of cases (%)	No. of cases (%)	
Age (years), median (IQR)	54 (46–63)	52 (44–62)	54 (46–63)	55 (46–64)	0.250
Initial 2014 FIGO stage					0.200
I–II	337 (44.2)	38 (37.6)	87 (42.0)	212 (46.6)	
III–IV	426 (55.8)	63 (62.4)	120 (58.0)	243 (53.4)	
Histologic type					0.499
Serous	434 (56.9)	59 (58.4)	125 (60.4)	250 (54.9)	
Mucinous	102 (13.4)	16 (15.8)	28 (13.5)	58 (12.7)	
Endometrioid	79 (10.4)	8 (7.9)	14 (6.8)	57 (12.5)	
Clear cell	96 (12.6)	12 (11.9)	24 (11.6)	60 (13.2)	
Others/NOS	52 (6.8)	6 (5.9)	16 (7.7)	30 (6.6)	
Grade					< 0.001*
Low	163 (21.4)	10 (9.9)	44 (21.3)	109 (24.0)	
High	462 (60.6)	31 (30.7)	107 (51.7)	324 (71.2)	
Unknown	138 (18.1)	60 (59.4)	56 (27.1)	22 (4.8)	
Timing of primary cytoreduction					< 0.001*
Primary debulking surgery	577 (75.6)	95 (94.1)	168 (81.2)	314 (69.0)	
Interval debulking surgery	154 (20.2)	5 (5.0)	35 (16.9)	114 (25.1)	
No surgery (only chemotherapy)	32 (4.2)	1 (1.0)	4 (1.9)	27 (5.9)	
Extent of primary cytoreduction^a^					< 0.001*
Complete	523 (71.5)	46 (46.0)	123 (60.6)	354 (82.7)	
Optimal (RT < 1 cm)	107 (14.6)	17 (17.0)	44 (21.7)	46 (10.7)	
Suboptimal (RT ≥ 1 cm)	87 (11.9)	29 (29.0)	32 (15.8)	26 (6.1)	
Unknown	14 (1.9)	8 (8.0)	4 (2.0)	2 (0.5)	
Secondary cytoreduction					0.563
Complete/optimal (RT < 1 cm)	82 (10.7)	9 (8.9)	26 (12.6)	47 (10.3)	
Suboptimal/unknown extent/no surgery	681 (89.3)	92 (91.1)	181 (87.4)	408 (89.7)	
Number of first‐line chemotherapy cycles					< 0.001**
≤ 6	577 (75.6)	92 (91.1)	159 (76.8)	326 (71.6)	
> 6	186 (24.4)	9 (8.9)	48 (23.2)	129 (28.4)	
Bevacizumab use					< 0.001***
Never	618 (81.0)	100 (99.0)	190 (91.8)	328 (72.1)	
First‐line	57 (7.5)	0 (0.0)	2 (1.0)	55 (12.1)	
≥ Second‐line	88 (11.5)	1 (1.0)	15 (7.2)	72 (15.8)	
PARPi use					< 0.001***
Never	703 (92.1)	101 (100)	203 (98.1)	399 (87.7)	
First‐line	11 (1.4)	0 (0.0)	0 (0.0)	11 (2.4)	
≥ Second‐line	49 (6.4)	0 (0.0)	4 (1.9)	45 (9.9)	
Follow‐up duration (months), median (IQR)	62.6 (37.6–98.2)	70.7 (28.6–177.1)	78.8 (37.6–134.1)	60.3 (40.4–80.0)	< 0.001***

Abbreviations: FIGO, The International Federation of Gynecology and Obstetrics; IQR, Interquartile range; NOS, not otherwise specified; PARPi, poly(ADP‐ribose) polymerase inhibitor; RT, residual tumor.

^a^ Only cases undergoing primary or interval debulking surgery were included.

* All pairwise comparisons were statistically significant.

** Group 1 differed significantly from the other groups.

*** Group 3 differed significantly from the other groups.

Procedures performed during primary cytoreduction are summarized in Table 2 to assess surgical radicality. No significant differences were observed in rates of hysterectomy, salpingo‐oophorectomy, omentectomy, small bowel resection, bladder/ureter resection, stomach resection, splenectomy, distal pancreatectomy, liver resection, or lung/pleural surgery. The rate of large bowel resection increased significantly from Group 1 to Group 2 but declined in Group 3 (12.0% vs. 26.6% vs. 18.7%; *p* = 0.007), as did cholecystectomy (0.0% vs. 5.9% vs. 3.5%; *p* = 0.035). Appendectomy rates decreased over time (66.0% vs. 34.5% vs. 26.2%), whereas pelvic (44.0% vs. 73.9% vs. 76.6%) and para‐aortic lymph node dissection (2.0% vs. 38.9% vs. 35.3%) increased compared to Group 1 (both *p* < 0.001). Rates of extra‐abdominal lymph node dissection (0.0% vs. 0.5% vs. 4.2%) and diaphragm peritonectomy/resection (3.0% vs. 7.4% vs. 13.6%) also rose in Group 3 (*p* = 0.005 and 0.002, respectively).

**TABLE 2:** Types of primary cytoreductive procedures performed.

Surgical procedure	Total	Group 1 (2003–2008)	Group 2 (2009–2013) with optimal cytoreduction	Group 3 (2014–2020) with bevacizumab ± PARPi	*p*
	*N* = 731^a^	*N* = 100 (13.7%)^a^	*N* = 203 (27.8%)^a^	*N* = 428 (58.5%)^a^	
	No. of cases (%)	No. of cases (%)	No. of cases (%)	No. of cases (%)	
Hysterectomy^b^	630/693 (90.9)	87/97 (89.7)	177/191 (92.7)	366/405 (90.4)	0.597
Unilateral/Bilateral salpingo‐oophorectomy	730 (99.9)	100 (100)	202 (99.5)	428 (100.0)	0.415
Omentectomy	667 (91.2)	93 (93.0)	181 (89.2)	393 (91.8)	0.435
Small bowel resection	15 (2.1)	1 (1.0)	7 (3.4)	7 (1.6)	0.276
Large bowel resection	146 (20.0)	12 (12.0)	54 (26.6)	80 (18.7)	0.007*
Bladder/ureter resection	11 (1.5)	1 (1.0)	5 (2.5)	5 (1.2)	0.466
Appendectomy	248 (33.9)	66 (66.0)	70 (34.5)	112 (26.2)	< 0.001**
Pelvic lymph node dissection	522 (71.4)	44 (44.0)	150 (73.9)	328 (76.6)	< 0.001**
Para‐aortic lymph node dissection	232 (31.7)	2 (2.0)	79 (38.9)	151 (35.3)	< 0.001**
Extra‐abdominal lymph node dissection	19 (2.6)	0 (0.0)	1 (0.5)	18 (4.2)	0.005***
Diaphragm peritonectomy/resection	76 (10.4)	3 (3.0)	15 (7.4)	58 (13.6)	0.002****
Stomach resection	6 (0.8)	0 (0.0)	4 (2.0)	2 (0.5)	0.104
Splenectomy	21 (2.9)	0 (0.0)	9 (4.4)	12 (2.8)	0.094
Distal pancreatectomy	13 (1.8)	1 (1.0)	5 (2.5)	7 (1.6)	0.717
Liver resection	40 (5.5)	1 (1.0)	14 (6.9)	25 (5.8)	0.092
Cholecystectomy	27 (3.7)	0 (0.0)	12 (5.9)	15 (3.5)	0.035*
Lung/pleural surgery	1 (0.1)	0 (0.0)	0 (0.0)	1 (0.2)	1.000

^a^ Only cases undergoing primary or interval debulking surgery were included.

^b^ Cases with a previous history of hysterectomy were excluded from percentage calculations.

* Group 1 differed significantly from Group 2.

** Group1 differed significantly from the other groups.

*** Group 2 differed significantly from Group 3.

**** Group 1 differed significantly from Group 3.

**FIGURE 1:**
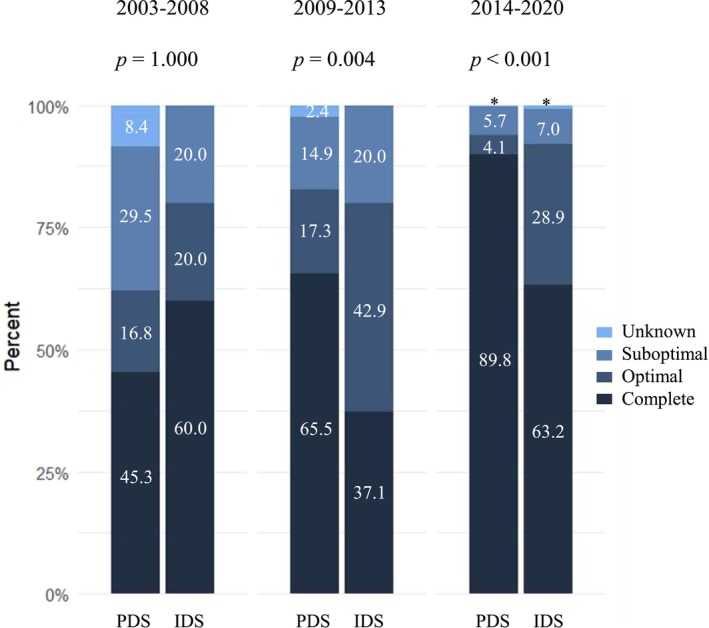
Proportions of debulking surgery extent stratified by timing of surgery and year of diagnosis. The association between timing and extent of surgery was analyzed using the Fisher test. *Percentages of unknown extent of debulking surgery in 2014–2022 were 0.3% for PDS and 0.9% for IDS, respectively. IDS, interval debulking surgery; PDS, primary debulking surgery.

After univariable Cox analyses, variables including age, year of diagnosis, initial FIGO stage, histologic type and grade, timing and extent of primary cytoreduction, and bevacizumab use were incorporated into the multivariable model (Table 3). In multivariable Cox analyses, poorer OS was associated with advanced stage (HR 10.23, 95% CI 5.03–20.83; *p* < 0.001), certain histologic types (mucinous: HR 6.28, 95% CI 3.29–11.98; clear cell: HR 3.30, 95% CI 1.69–6.44; both *p* < 0.001), optimal debulking in IDS with residual disease (HR 2.94, 95% CI 1.49–5.79; *p* = 0.002), and suboptimal/unknown extent/no surgery (HR 4.06, 95% CI 2.46–6.71; *p* < 0.001). Complete resection during PDS was significantly higher than IDS in both Group 2 (PDS: 65.3% vs. IDS: 37.1%; *p* = 0.004) and Group 3 (PDS: 89.8% vs. IDS: 63.2%; *p* < 0.001) (Figure 1).

**TABLE 3:** Univariable and multivariable Cox regression analyses for overall survival in patients with epithelial ovarian cancer.

Variable	*N* (%)	No. of deaths (%)	Univariable		Multivariable	
			HR (95% CI)	*p*	HR (95% CI)	*p*
Age (years)				0.001		
< 40	84 (11.0)	9 (10.7)	Ref		Ref	
40–49	188 (24.6)	27 (14.4)	1.26 (0.59–2.67)	0.553	1.16 (0.53–2.55)	0.712
50–59	218 (28.6)	35 (16.1)	1.50 (0.72–3.12)	0.28	1.28 (0.60–2.74)	0.525
60–69	161 (21.1)	22 (13.7)	1.38 (0.64–3.00)	0.415	0.88 (0.39–1.98)	0.748
≥ 70	112 (14.7)	25 (22.3)	3.27 (1.52–7.02)	0.002	1.73 (0.75–3.97)	0.199
Year of diagnosis				0.057		
2003–2008	101 (13.2)	19 (18.8)	Ref		Ref	
2009–2013	207 (27.1)	45 (21.7)	1.10 (0.64–1.88)	0.734	1.72 (0.98–3.00)	0.059
2014–2020	455 (59.6)	54 (11.9)	0.69 (0.41–1.16)	0.162	1.17 (0.67–2.05)	0.558
Initial 2014 FIGO stage				< 0.001		
I–II	337 (44.2)	13 (3.9)	Ref		Ref	
III–IV	426 (55.8)	105 (24.6)	9.03 (5.07–16.09)	< 0.001	10.23 (5.03–20.83)	< 0.001*
Histologic type				0.003		
Serous	434 (56.9)	76 (17.5)	Ref		Ref	
Mucinous	102 (13.4)	15 (14.7)	0.93 (0.53–1.62)	0.795	6.28 (3.29–11.98)	< 0.001*
Endometrioid	79 (10.4)	3 (3.8)	0.19 (0.06–0.59)	0.004	0.85 (0.26–2.75)	0.785
Clear cell	96 (12.6)	12 (12.5)	0.65 (0.36–1.20)	0.172	3.30 (1.69–6.44)	< 0.001*
Others	52 (6.8)	12 (23.1)	1.62 (0.88–2.98)	0.121	1.38 (0.75–2.57)	0.302
Grade				0.004		
Low	163 (21.4)	12 (7.4)	Ref		Ref	
High	462 (60.6)	79 (17.1)	2.52 (1.37–4.62)	0.003	0.98 (0.46–2.11)	0.968
Unknown	138 (18.1)	27 (19.6)	2.93 (1.48–5.79)	0.002	0.97 (0.44–2.16)	0.940
Primary cytoreduction (timing and extent)				< 0.001		
Complete/Optimal debulking in PDS (RT < 1 cm)	493 (64.6)	41 (8.3)	Ref		Ref	
Complete in IDS	88 (11.5)	18 (20.5)	3.02 (1.73–5.26)	< 0.001	1.58 (0.86–2.92)	0.144
Optimal debulking in IDS (RT < 1 cm)	49 (6.4)	13 (26.5)	5.79 (3.09–10.85)	< 0.001	2.94 (1.49–5.79)	0.002*
Suboptimal/unknown extent/no surgery	133 (17.4)	46 (34.6)	7.25 (4.74–11.08)	< 0.001	4.06 (2.46–6.71)	< 0.001*
Secondary cytoreduction				0.406		
Complete/optimal debulking (RT < 1 cm)	82 (10.7)	13 (15.9)	Ref		—	—
Suboptimal/unknown extent/no surgery	681 (89.3)	105 (15.4)	1.28 (0.72–2.28)	0.407	—	—
Bevacizumab				< 0.001		
Never	618 (81.0)	82 (13.3)	Ref		Ref	
First‐line	57 (7.5)	14 (24.6)	2.66 (1.50–4.71)	< 0.001	1.06 (0.55–2.04)	0.861
≥ Second‐line	88 (11.5)	22 (25.0)	2.00 (1.25–3.21)	0.004	1.30 (0.76–2.21)	0.341
PARPi use				0.384		
Never	703 (92.1)	110 (15.6)	Ref		—	—
First‐line	11 (1.4)	0 (0.0)	NA	0.954	—	—
≥ Second‐line	49 (6.4)	8 (16.3)	0.84 (0.41–1.72)	0.635	—	—

Abbreviations: CI, confidence interval; FIGO, The International Federation of Gynecology and Obstetrics; HR, hazard ratio; IDS, interval debulking surgery; PARPi, poly(ADP‐ribose) polymerase inhibitor; PDS, primary debulking surgery; RT, residual tumor.

* Significantly different.

**FIGURE 2:**
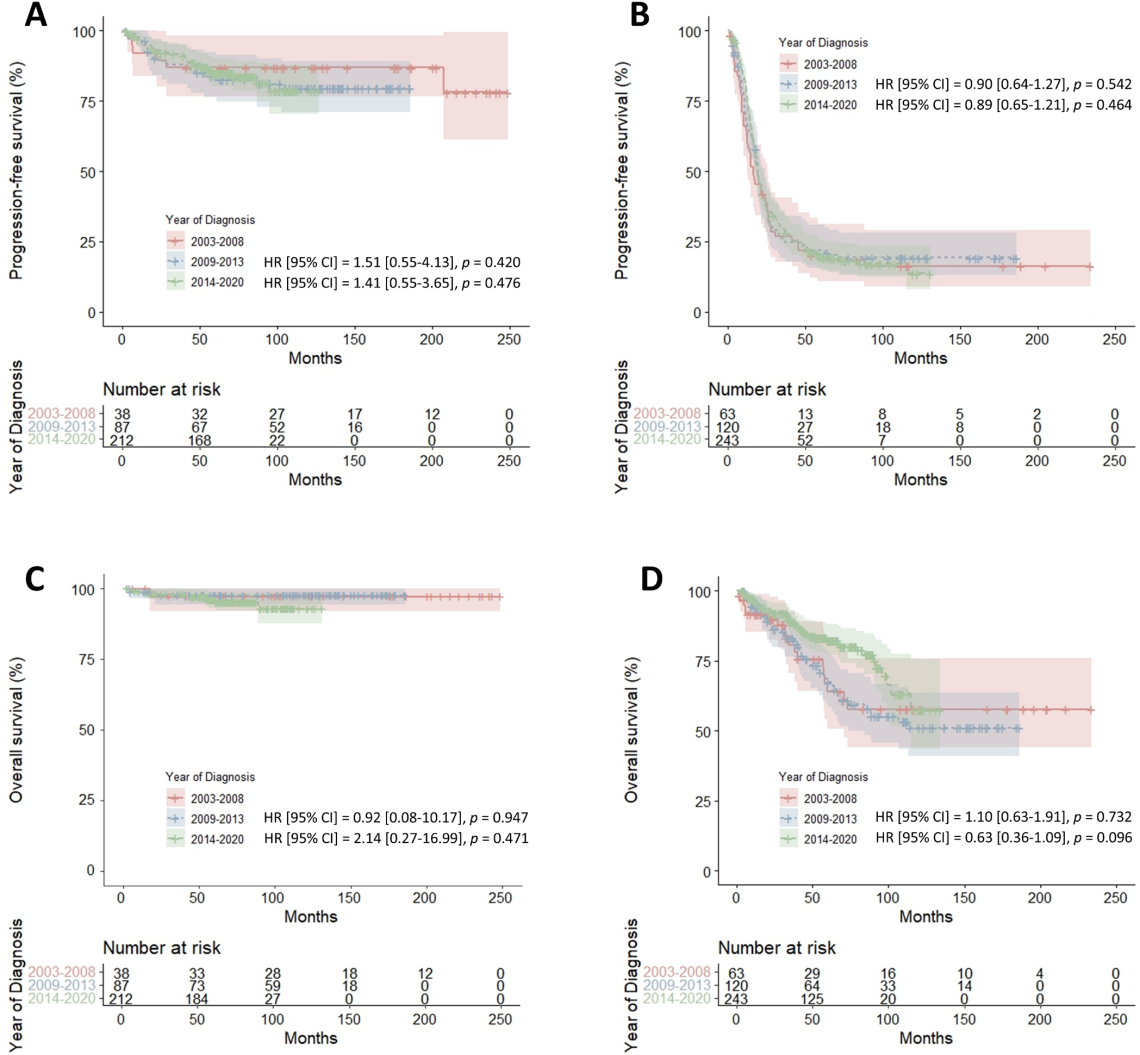
Progression‐free survival (PFS) at first recurrence and overall survival (OS) by initial stage (2014 FIGO) and year of diagnosis: (A) PFS in Stage I–II, (B) PFS in Stage III–IV, (C) OS in Stage I–II, and (D) OS in Stage III–IV.

Table 4, based on the log‐rank test, shows no significant difference in 5‐year PFS at first recurrence among the three groups for either early‐ or advanced‐stage patients (Figure 2A,B). In contrast, 5‐year OS improved significantly in FIGO Stage III–IV patients from 2009–2014 to 2015–2020 (67.3% vs. 82.5%; *p* = 0.038), but not in early‐stage patients (Figure 2C,D). However, this improvement was not statistically significant by HR analysis (HR 0.63, 95% CI 0.36–1.09; *p* = 0.096). The time‐dependent HR to assess whether the effect of diagnosis year on survival changed over time found no statistically significant change (HR 1.19, 95% CI 0.90–1.57; *p* = 0.217).

**TABLE 4:** Five‐year progression‐free survival at first recurrence and overall survival by initial stage (2014 FIGO) and diagnosis period in patients with epithelial ovarian cancer (log‐rank test).

Variable	Progression‐free survival		Overall survival	
	5‐year (%)	*p*	5‐year (%)	*p*
FIGO I–II		0.716		0.457
2003–2008	86.8		97.2	
2009–2013	84.0		97.6	
2014–2020	84.8		95.9	
FIGO III–IV		0.758		0.024*
2003–2008	18.3		64.0	
2009–2014	21.9		67.3	
2015–2020	19.7		82.5	
All stages		0.459		0.057
2003–2008	44.6		79.2	
2009–2013	47.6		80.9	
2014–2020	49.8		89.4	

Abbreviation: FIGO, The International Federation of Gynecology and Obstetrics.

* A significant difference was observed between 2009–2014 and 2015–2020 in FIGO Stage III–IV by post hoc analysis (*p* = 0.038).

## 4 Discussion

Through a review of two decades of clinical practice, we observed that major advancements in ovarian cancer management have been integrated into real‐world settings, although the use of PARP inhibitors remained low at 12.3%. During this period, OS for advanced disease significantly improved, whereas PFS remained largely unchanged. In our cohort, advanced stage, aggressive histologic subtypes, and the absence of PDS with optimal cytoreduction or IDS with complete resection were associated with poorer OS.

The timing and extent of primary cytoreduction differed among groups stratified by diagnosis year. Although both IDS rates and optimal cytoreduction rates increased over time, unlike previous reports, the increasing likelihood of optimal debulking in our temporal cohort was not entirely attributable to the increased use of IDS (Figure 1) [17]. Similarly, analysis of the US National Cancer Database showed increased neoadjuvant chemotherapy use since the 2000s, accompanied by improvements in OS, irrespective of treatment modality [18]. This likely reflects advances in multiple factors, including selection of appropriate surgeries, surgical skills and technology, chemotherapy regimens and other medications, and supportive care. In our cohort, this is evidenced by the increasing proportion of complete debulking in PDS, as well as the adoption of more extensive and radical procedures, such as diaphragm surgery and multi‐regional lymphadenectomy.

Concerns regarding IDS include potential association with platinum resistance, such as a higher likelihood of platinum‐resistant recurrence and a shorter interval to recurrence [19]. Several studies, including randomized phase III trials, have reported the noninferiority of IDS compared with PDS in advanced ovarian cancer [20, 21, 22]. However, a Japanese trial comparing 152 IDS with 149 PDS patients did not confirm this (HR 1.05, 90.8% CI 0.83–1.33) [23]. In our study, IDS with optimal debulking (excluding complete resection) was associated with poorer OS compared with PDS (HR 2.94, 95% CI 1.49–5.79). It is essential to consider not only the variables being compared but also differences in study design, treatment protocols, and surgical outcomes. For instance, the Japanese trial allowed crossover (33.3% of PDS patients underwent IDS) and included patients under 75 years old with Stage III/IV disease. Notably, complete/optimal surgery rates were lower in that trial (PDS: 38%, IDS: 82%) than in our cohort (PDS: 66.1% [160/242], IDS: 88.8% [135/152] among advanced cases). In contrast, the international randomized multicenter phase III TRUST trial, conducted at expert centers, reported complete resection rates of 62.9% for PDS and 76.6% for IDS [24]. It concluded that PDS resulted in significantly longer median PFS and numerically longer OS than IDS in non‐frail patients, highlighting the importance of complete resection rates.

Additionally, a U.S. retrospective cohort study suggested that defining IDS as optimal with residual disease < 1 cm may be inadequate when multiple anatomic sites are involved [25]. In our comparison of IDS cases—complete resection versus residual disease < 1 cm—with optimal PDS cases (complete resection or residual disease < 1 cm), complete resection in IDS was not statistically significant, whereas IDS with any residual disease, even < 1 cm, was associated with poorer OS. Although IDS remains valuable for patients medically unsuitable for or surgically unresectable at PDS, caution is warranted regarding residual disease.

We initially aimed to assess oncologic outcomes based on major changes, including extensive surgery and maintenance therapy. However, maintenance therapy use was relatively limited (27.9% bevacizumab, 12.3% PARP inhibitors in Group 3). Therefore, although there have been some recent improvements in OS, the contributing factors remain unclear. These may include extensive surgery or its timing, maintenance therapies, chemotherapy, supportive care, or combinations of these factors.

Notably, PFS after primary treatment showed no improvement, regardless of initial stage or year of diagnosis. Considering the reduced tumor burden following primary optimal debulking surgery, this may nevertheless influence prognosis after the first recurrence. In our cohort, bevacizumab and PARP inhibitors were used primarily in second‐ or later‐line therapies rather than as first‐line treatments. Other important factors, including active and enhanced salvage therapies and appropriate supportive care, should also be taken into account. Given that improvements were observed only in OS within the evolving treatment paradigm, future studies are needed to clarify the contributions of individual factors.

This study included early‐stage patients to compare OS, which may reflect the effects of treatment for recurrent disease. However, PFS and OS in this group did not improve compared with earlier periods, likely reflecting essentially unchanged treatment strategies. Efforts to improve ovarian cancer outcomes have primarily focused on advanced‐stage disease, which is understandable given its poor prognosis [26]. Although early‐stage patients generally have a better prognosis, some still experience recurrence and receive treatments similar to those for advanced disease. Among 55 (16.3%) recurrent early‐stage patients, 14 (25.5%) received ≥ second‐line bevacizumab, 6 (10.9%) received ≥ second‐line PARP inhibitors, and 26 (47.3%) underwent secondary complete or optimal cytoreduction. Greater adoption of recent therapeutic advances may improve outcomes and address residual mortality in this population.

Previous studies evaluating temporal survival trends in EOC have demonstrated favorable outcomes, supporting the importance of active treatment. Incorporating extensive upper abdominal procedures increased optimal cytoreduction rates and significantly improved both PFS and OS [27]. Before the introduction of targeted therapy, an analysis of the Korea Central Cancer Registry reported a 5‐year relative survival rate improvement from 57.2% in 1995–1999 to 63.8% in 2010–2014 [28]. Similarly, an analysis of the Surveillance, Epidemiology, and End Results database (1990–2014) showed a decline in surgery use (92.0% to 88.9%) and an increase in chemotherapy (67.4%–75.0%) for distant‐stage disease [29]. Over the same period, 5‐year cause‐specific survival (CSS) and OS improved slightly in localized disease (CSS: 91.9% to 93.1%; OS: 85.6% to 88.5%) and markedly in distant disease (CSS: 31.4%–42.7%; OS: 26.7%–37.4%). Survival improvements in EOC occurred before 2014, preceding the widespread adoption of targeted therapies. However, in our non‐population‐based cohort, we observed increased survival only among patients diagnosed after 2014, not before.

Meanwhile, data from the Netherlands Cancer Registry (1989–2014) showed slight improvements in 10‐year survival for early‐stage (62%–67%) and advanced‐stage disease (10%–13%), with OS for all patients essentially unchanged at 24%, despite increased use of optimal treatment [30]. The authors concluded that long‐term survival in women with EOC has not improved over the past 25 years, suggesting that observed gains in 5‐year OS likely reflect improved staging and prolonged disease control rather than increased cure rates. Similarly, in our cohort, the only improved oncologic outcome—5‐year survival in advanced‐stage patients—lost significance in the Cox analysis, as the survival curves converged after approximately 100 months (Figure 2D). Considering that the time‐dependent analysis indicated no significant change in hazard over time, this may be due to censoring and the reduced number of events in the later follow‐up. In our cohort, OS for all patients showed a numerical improvement, though not statistically significant (OS: 79.2% in 2003–2008 vs. 89.4% in 2014–2020; *p* = 0.057).

Several limitations should be considered. First, this single‐center cohort in South Korea had a relatively small sample size, which may limit generalizability despite generally uniform clinical practices under the national insurance system. Observed improvements in oncologic outcomes could reflect not only changes in the analyzed therapies but also accumulated experience at the center. Second, the retrospective design introduces potential selection and information bias. Group classification based on treatment trends led to imbalances, particularly the relatively small size of Group 1 and the shorter follow‐up in Group 3, which may create heterogeneity and limit the accuracy of comparisons. For example, the larger sample size of Group 3 may include more favorable patients, and its shorter follow‐up could overestimate OS due to missed late recurrences. However, as the convergence of the survival curves in Figure 2D may reflect censoring, longer follow‐up could reveal a more definitive survival benefit. Given these conflicting possibilities and the potential for late recurrence in ovarian cancer, further studies with follow‐up beyond 5 years are warranted.

In addition, histologic grading of IDS cases based on surgical specimens may have been affected by prior chemotherapy. Mortality data were obtained from institutional records rather than a national database, potentially leading to underreporting. Precise PFS assessment was limited by the retrospective nature and, to some extent, changes in diagnostic criteria [16]. Outcomes may also have been influenced by unmeasured confounders, such as chemotherapy regimens and BRCA mutation status. Weekly paclitaxel combined with triweekly carboplatin has been associated with improved outcomes compared with the triweekly regimen, although this benefit has not been consistently replicated [31, 32, 33]. Additionally, patients with BRCA mutations tend to have a better prognosis and enhanced responses to therapies, including PARP inhibitors; however, our cohort includes patients diagnosed as early as 2003, when genetic testing was not routinely performed [34]. A prospective multicenter study of Korean women with high‐grade serous and/or endometrioid EOC diagnosed between 2010 and 2015 reported germline BRCA mutations in 26.2% (78/298) and somatic BRCA mutations in 12.8% (11/86) of those without germline BRCA mutations [35]. These prevalence estimates were relatively higher than previously reported in other ethnic groups.

In conclusion, clinical practices have evolved alongside numerous advances and changes in ovarian cancer treatment, including increased adoption of IDS, higher rates of complete debulking in PDS, and more first‐line chemotherapy cycles. Following the widespread introduction of targeted therapies, patients diagnosed during this period demonstrated improved outcomes. However, these gains likely reflect cumulative efforts across multiple treatment modalities rather than the effects of targeted or maintenance therapies alone, particularly given their relatively limited adoption to date. Despite these advances, advanced ovarian cancer remains associated with a poor prognosis, highlighting the need for continued efforts to optimize surgical strategies—such as appropriate radicality and timing—and to develop more effective systemic therapies. Real‐world data suggest that these improvements translate into higher 5‐year OS among patients recently diagnosed with advanced‐stage disease. Future studies should further clarify the impact of widespread real‐world adoption of treatments validated in individual studies, including targeted therapies, with particular emphasis on long‐term evaluation.

## Author Contributions

**Jeongyun Kim:** conceptualization, investigation, writing – original draft, writing – review and editing, visualization, methodology, formal analysis, software, data curation, validation. **Dong Hoon Suh:** writing – review and editing, resources. **Kidong Kim:** writing – review and editing, resources. **Jae Hong No:** writing – review and editing, resources. **Yong Beom Kim:** conceptualization, funding acquisition, writing – review and editing, project administration, resources, methodology, software, data curation, supervision.

## Funding

This study was supported by Samyang Holdings Corp., during the preparation of the manuscript.

## Disclosure

The authors have nothing to report.

## Ethics Statement

The Institutional Review Board of Seoul National University Bundang Hospital approved this study (IRB No. B‐2404‐897‐106) and waived the need for informed consent because of its retrospective nature and use of de‐identified data.

## Consent

No written consent has been obtained from the patients as there is no patient identifiable data included.

## Conflicts of Interest

The authors declare no conflicts of interest.

## Data Availability

The data supporting the findings of this study are not publicly accessible because of institutional policies and ethical requirements at the participating facilities. Data may be shared upon reasonable request, subject to approval from the institutional review boards of Seoul National University Bundang Hospital.
